# Notes and Notices

**Published:** 1894-06

**Authors:** 


### NOTES AND NOTICES.

THE INTERNATIONAL HAHNEMANNIAN ASSOCIATION will hold its next annual meeting at Niagara Falls, probably about June 26th. There has been some objection made to the previous date, and a vote was taken to decide whether that or some other date should be fixed.

DR. THOMAS SKINNER has removed from 25 Somerset Street, Portman Square, W., to 6 York Place, Portman Square, W., London, England.

